# Oxidized regenerated cellulose particles enable high-pressure air-leak sealing on lung segmentectomy surfaces in porcine models

**DOI:** 10.3389/fbioe.2026.1789890

**Published:** 2026-07-15

**Authors:** Qianrui Ren, Kun Wang, Wenbo Sun, Zhibo Wang, Xinfeng Xu, Wei Wen, Quan Zhu

**Affiliations:** Department of Thoracic Surgery, The First Affiliated Hospital of Nanjing Medical University, Nanjing, Jiangsu, China

**Keywords:** fibrin glue, lung segmentectomy, oxidized regenerated cellulose particles, PGA sheet, pulmonary air leakage, sealing efficacy

## Abstract

**Objective:**

Pulmonary air leakage is a common complication that hinders rapid recovery after lung segmentectomy. Oxidized regenerated cellulose flat (ORCF) combined with fibrin glue (FG) has shown efficacy in preventing air leaks in previous studies but is limited by inadequate tissue coverage. This study aimed to evaluate commercially available oxidized regenerated cellulose particles (ORCP; SURGICEL Powder) using a porcine model of lung segmentectomy to address the limitations of conventional ORCF.

**Methods:**

A porcine lung segmentectomy model was established, and experimental animals were randomly assigned to six groups: blank control, FG alone, ORCF-FG, polyglycolic acid (PGA) sheet-FG, ORCP-FG, and ORCP-FG-ORCF. The sealing efficacy of different treatments was evaluated by measuring the minimum air leakage pressure (MALP). Histological characteristics of the sealed resection surfaces were further analyzed, including quantitative assessment of interfacial gap ratio and sealing-layer thickness.

**Results:**

The ORCP-FG-ORCF group achieved the highest MALP (101.5 ± 5.2 cmH_2_O), which was significantly higher than that of all other groups (p < 0.001). The ORCP-FG and PGA-FG groups exhibited comparable MALP values, both significantly higher than that of the ORCF-FG group. Histological analysis demonstrated improved sealing-layer integration and adherence in the ORCP-FG and PGA-FG groups relative to the ORCF-FG group. Quantitative histological analysis further revealed that the PGA-FG group had the lowest interfacial gap ratio, while sealing-layer thickness did not parallel mechanical sealing performance across groups.

**Conclusion:**

ORCP-FG exhibits strong sealing performance in this porcine ex vivo model, with efficacy comparable to the current clinical benchmark of PGA-FG. As an improved biodegradable sealing material, ORCP overcomes the insufficient tissue coverage of conventional flat ORCF, serving as a mechanically effective alternative for preventing postoperative pulmonary air leakage after lung segmentectomy and providing a promising new clinical strategy.

## 1 Introduction

The JCOG0802/WJOG4607L trial conducted in Japan has demonstrated that segmentectomy yields significantly better overall survival than lobectomy in patients with stage IA non-small cell lung cancer. This indicates that segmentectomy has the potential to become the standard surgical approach for early-stage lung cancer (Saji et al., 2022). However, the segmentectomy group exhibited a higher incidence of postoperative pulmonary air leakage (6.5% vs. 3.8%, p = 0.04), particularly among patients undergoing complex segmentectomies or those with a history of long-term smoking (Suzuki et al., 2019). Therefore, addressing postoperative pulmonary air leakage has become a critical clinical challenge. Current evidence shows that the combination of polyglycolic acid (PGA) sheets and fibrin glue (FG) can effectively seal pleural defects and reduce air leakage following segmentectomy (Gika et al., 2007). Nevertheless, the clinical application of PGA sheets is limited by their high cost, inflammatory responses after degradation (Ceonzo et al., 2006; Yang et al., 2005) and the risk of pleural adhesions (Nakamura et al., 2010; Saito et al., 2017)Consequently, research on finding alternative materials is ongoing.

Oxidized regenerated cellulose is a bioabsorbable material derived from the controlled oxidation of cellulose. In a prior study, we developed a technique that uses ORC flat (ORCF) combined with FG to cover the resection surface during segmentectomy, aiming to prevent postoperative pulmonary air leakage (Yang et al., 2022). A porcine segmentectomy model was also established to evaluate the adhesion strength and efficacy in reducing air leakage. The results indicated that the combination of ORCF and FG can meet the basic requirements for early postoperative airway pressure tolerance. It also has advantages such as lower cost, excellent hemostatic properties, and favorable biocompatibility (Lewis et al., 2013; Martyn et al., 2015) demonstrating promising clinical potential. However, the sealing performance of this combination is still inferior to that of PGA sheets combined with FG.

In this study, we evaluated commercially available absorbable oxidized regenerated cellulose hemostatic particles (ORCP; SURGICEL Powder). These particles are composed of ORC in an aggregated particulate structure (Wang et al., 2017). We hypothesize that this structural characteristic enables better adaptation to the irregular resection surfaces during segmentectomy. By incorporating ORCP into the pleural coverage procedure, we aim to enhance the sealing efficacy of FG. To maintain methodological consistency, we used the same experimental protocol to assess the air leakage prevention capacity of the ORCP-FG combination and performed histological examinations to determine tissue adhesion strength. This study seeks to investigate whether ORCP used in conjunction with FG can improve mechanical air-leak sealing after segmentectomy.

## 2 Materials and methods

### 2.1 Porcine lungs

Bilateral porcine lungs were obtained from healthy domestic pigs aged approximately 6 months and weighing (100 ± 25) kg. The lungs were sourced post-slaughter on the same day from Jiangsu Jurong Kangrong Poultry Industry Co., Ltd. Lungs exhibiting large bullae, severe congestion, infection, trauma, anatomical variations, or other abnormalities that could potentially compromise experimental outcomes were excluded. To preserve pulmonary physiological integrity and minimize loss of pulmonary surfactant, all experiments were conducted within 4 h of lung excision.

### 2.2 Sealing materials

Fibrin Glue (Beixiu®, Porcine Fibrin Adhesive; Guangzhou, China) is a synthetic absorbable biomaterial composed of two components: a main glue containing fibrinogen and coagulation factor XIII, and a catalytic solution containing thrombin and calcium chloride (CaCl_2_). Upon mixing, these solutions simulate the final phase of the coagulation cascade. This process forms a stable fibrin polymer with a cross-linked, uniform network structure. This mesh-like matrix immobilizes erythrocytes and plasma components, serves as a biological scaffold for fibroblast migration and capillary ingrowth, and promotes pleural sealing and repairing. Additionally, as a flexible mechanical barrier against air leakage, it remains functional for up to 14 days, allowing sufficient time for tissue expansion and wound healing. Fibrin glue has excellent biocompatibility, and its degradation products are safely excreted through renal metabolism.

**FIGURE 1:**
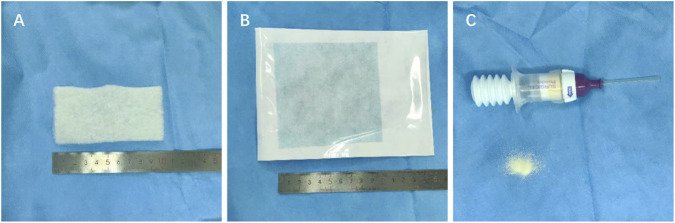
**(A)** ORC flat; **(B)** absorbable PGA sheet; **(C)** absorbable ORC hemostatic particles. ORC (oxidized regenerated cellulose), PGA (polyglycolic acid).

Oxidized Regenerated Cellulose Flat (Ethicon Surgicel® Absorbable Hemostat; Johnson & Johnson, New Jersey, USA) can be trimmed to fit irregular surfaces (Figure 1A). Its fabric-like texture allows good conformity to tissue contours, ensuring close contact. As a cellulose derivative, ORCF have favorable biocompatibility, biodegradability and low toxicity. Their hemostatic mechanism involves both physical and chemical actions. Physically, the mesh structure promotes platelet adhesion and aggregation. It enhances the conversion of fibrinogen to fibrin, thereby facilitating thrombus formation. Chemically, hydroxyl groups in cellulose react with Fe^2+^ in plasma. This reaction forms a dark gelatinous acid-hematin complex, which aids hemostasis (Wu et al., 2012). Furthermore, ORCF is nearly completely absorbed within 7 days and does not cause adverse tissue reactions.

Absorbable PGA Sheet (Neville®, Okazawa Co., Ltd., Kyoto, Japan) is constructed from a loose, highly elastic PGA fiber matrix, with a fiber diameter of 16.1 nm, an average inter-fiber distance of 27.4 nm, and an average thickness of 0.15 mm (Figure 1B). Available in tubular and flake forms, Neville® was used in flake form in this study. The material is fully absorbed *in vivo* within approximately 15 weeks.

Absorbable Oxidized Regenerated Cellulose Hemostatic Particles (SURGICEL Powder and SURGICEL Endoscopic Applicator; Johnson & Johnson, New Jersey, USA) are hemostatic materials composed of compacted fine ORC fibers formed through a proprietary manufacturing process (Figure 1C). Their aggregated particulate structure enables tight adherence to bleeding surfaces and rapid penetration into blood layers to exert hemostatic effects. ORCP have demonstrated efficacy in controlling mild to moderate hemorrhage while retaining the advantageous biochemical properties of standard ORC materials. When used appropriately, they are largely absorbed within 2–5 weeks and do not induce significant local adverse reactions (Al-Attar et al., 2023; MacDonald et al., 2017).

### 2.3 Surgical procedure

**FIGURE 2:**
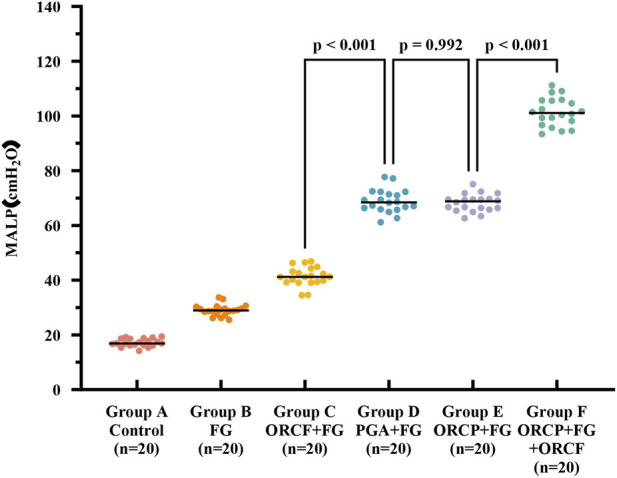
Anatomical diagram of porcine lung. R1: right apical lobe; R2: right cardiac lobe; R3: right diaphragmatic lobe; R4: right accessory lobe; L1: left apical lobe; L2: left diaphragmatic lobe. R1a, R1b (a and b segments of the right apical lobe); L1a, L1b (a and b segments of the left apical lobe). In this study, the R1a subsegment was selected for pulmonary segmentectomy, and a resection plane was established between R1a and R1b.

The experimental procedure was conducted strictly in accordance with the methods and parameters established in our previous study. Based on prior findings, the surgical model using the A segment (R1a segment) of the right apical lobe in porcine lungs requires a relatively short operative duration. This efficiency is likely attributable to the anatomical feature that the R1a segmental bronchus arises directly from the main bronchus (Figure 2), thereby simplifying dissection and resection. Furthermore, no significant differences in experimental outcomes have been observed across models utilizing different pulmonary segments (Yang et al., 2022). To optimize procedural efficiency without compromising reproducibility or validity, the R1a segment was selected for use in this study to establish the porcine lung segmentectomy model. After bronchial suctioning, a 12-Fr cuffed endotracheal tube was connected to a mechanical ventilator (Aestiva/5 7, 100, Datex-Ohmeda, Madison, Wisconsin, USA). The distal end of the tube was inserted into the target segmental bronchus. The cuff was inflated to ensure an airtight seal. The lung specimen was then submerged in warmed normal saline (37 °C), and the target segment was gradually inflated using a stepwise airway pressure method. Air leakage pressure was measured with a micromanometer (BENETECH, Jumaoyuan Technology Co., Ltd., Shenzhen, China, GM520, ±35 kPa, ±0.3%). Inflation pressure was maintained below 20 cmH_2_O to prevent alveolar overdistension. If adequate inflation could not be achieved or significant air leakage occurred, the specimen was excluded from further analysis.

A monopolar electrocautery device (POWER-420B, Changzhou Yanling Electronic Equipment Co., Ltd., Changzhou, China) was used at a power setting of 40 W to separate the intersegmental plane. Specimens with incorrect segmentation were discarded. Connective tissues and hilar vessels were incised using the scalpel, and the target bronchus was finally cut off to complete the segmentectomy.

### 2.4 Measurement of pulmonary air leak pressure

Lung segments were immersed in a 37 °C physiological saline bath and ventilated at an initial airway pressure of 10 cmH_2_O. Ventilation pressure was increased stepwise by 5 cmH_2_O per minute until reaching 70 cmH_2_O, the ventilator’s maximum capacity. The ventilator was set to pressure control mode, with parameters as follows: respiratory rate 12 breaths/min, inspiratory-to-expiratory ratio 1:2, and positive end-expiratory pressure 0 cmH_2_O. Intratracheal pressure was monitored through the endotracheal tube using the ventilator-integrated pressure sensor. Minimum air leakage pressure (MALP) was defined as the pressure at which continuous bubbles emerged from the submerged resection surface. If no leakage occurred at the 70 cmH_2_O ventilator setting, additional pressure was manually applied up to 70 cmH_2_O using a self-inflating ventilation bag, at a rate of 2 cmH_2_O/min until leakage was detected. Two investigators independently recorded MALP values. To eliminate observer bias, both investigators were strictly blinded to group allocations during the testing and data acquisition process. When the difference between their measurements exceeded 1 cmH_2_O, the test was repeated. If the discrepancy persisted after retesting, the lung segment was excluded from analysis. The final MALP for each segment was calculated as the average of the two recorded values.

### 2.5 Random allocation and sample size determination

The sample size was determined prior to the study based on an *a priori* power analysis. Assuming a two-sided Alpha (α) of 0.05 and a Power (1-β) of 80%, and expecting a large effect size (Cohen’s d = 1.0) regarding the difference in MALP between the materials based on previous comparable studies (Yang et al., 2022), the minimum required sample size was calculated to be 16 specimens per group. To conservatively account for the inherent anatomical variations of *ex vivo* porcine lung tissues and potential technical exclusions during preparation, we proactively increased the sample size by 25%, resulting in a final standardized cohort of n = 20 per group.

**FIGURE 3:**
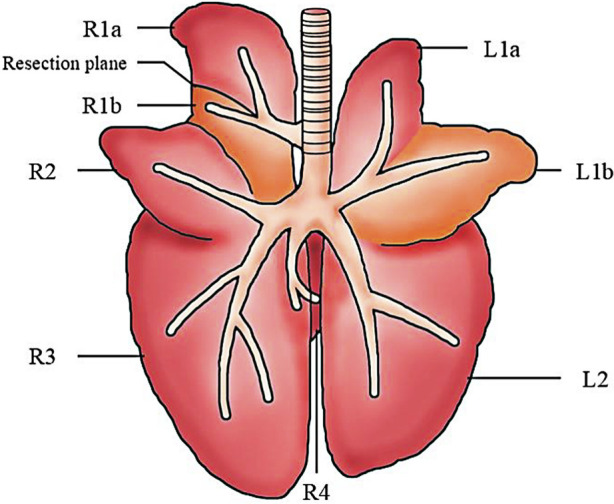
The procedures of sealing material application in each group.

To minimize selection bias, porcine lung segmentectomy models were randomly assigned to six experimental groups (n = 20 per group). Group A: No intervention was performed on the resection surface. Group B: 0.5 mL of liquid glue was sprayed onto the resection surface, followed by 0.5 mL of liquid catalyst. Group C: 0.25 mL of liquid main glue was sprayed onto the resection surface, then an ORCF (5.1 × 10.2 cm^2^, 2 mm thick) was placed over the interface after cutting off the extra parts of the periphery. Subsequently, 0.25 mL of liquid catalyst solution was sprayed onto the sheet, followed by an additional 0.25 mL of liquid main glue and 0.25 mL of liquid catalyst. Group D: 0.25 mL of liquid main glue was sprayed onto the resection surface, then a PGA sheet (100 × 50 mm^2^, 0.15 mm thick) was placed over the interface after cutting off the extra parts of the periphery. Subsequently, 0.25 mL of liquid catalyst solution was sprayed onto the sheet, followed by an additional 0.25 mL of liquid main glue and 0.25 mL of liquid catalyst. Group E: 0.25 mL of liquid main glue was sprayed onto the resection surface, followed by application of ORCP to the interface in three sequential doses (0.2 g each). Subsequently, 0.25 mL of liquid catalyst solution was sprayed onto the particles, followed by an additional 0.25 mL of liquid main glue and 0.25 mL of liquid catalyst. Group F: 0.25 mL of liquid main glue was sprayed onto the resection surface, followed by application of ORCP to the interface in three sequential doses (0.2 g each). Then an ORCF (5.1 × 10.2 cm^2^, 2 mm thick) was placed over the interface after cutting off the extra parts of the periphery. Subsequently, 0.25 mL of liquid catalyst solution was sprayed onto the particles, followed by an additional 0.25 mL of liquid main glue and 0.25 mL of liquid catalyst. The application procedures for sealing materials are illustrated in Figure 3. After material application, each specimen was allowed to stand for 5 min to ensure stabilization of the sealant. Thereafter, their MALPs were measured using the method mentioned above.

### 2.6 Histological examination

A 1 × 1 cm lung tissue sample, centered on the segmental resection plane and covered with the sealing material, was harvested for histological examination. Specimens were fixed in 10% neutral buffered formalin, processed for paraffin embedding, and sectioned for hematoxylin and eosin (H&E) staining. Each specimen was examined across at least five distinct histological sites to ensure comprehensive assessment.

To further improve the objectivity of histological evaluation, quantitative image analysis was performed using ImageJ software (National Institutes of Health, Bethesda, MD, USA). For each section, sealing layer thickness was measured as the perpendicular distance between the outer surface of the sealing layer and the pleural surface at selected representative points. In addition, the interfacial gap ratio was determined by calculating the proportion of visible gap area relative to the total material–tissue interface area. All measurements were conducted under identical magnification and imaging settings.

### 2.7 Statistical analysis

All statistical analyses were performed using SPSS 21.0 software (SPSS Inc., Chicago, IL, USA). Data were expressed as mean ± standard deviation. A p-value <0.05 was considered statistically significant. Homogeneity of variance was assessed using Levene’s test, which indicated a significant violation of variance homogeneity across groups (W = 5.965, p < 0.001). However, the Shapiro-Wilk test confirmed that the data approximated a normal distribution (all p > 0.05). Given these conditions, Welch’s one-way ANOVA was employed to evaluate overall group differences, and the Games-Howell *post hoc* procedure was applied for pairwise multiple comparisons.

## 3 Results

### 3.1 Pressure resistance performances of the porcine lung segmentectomy models

**FIGURE 4:**
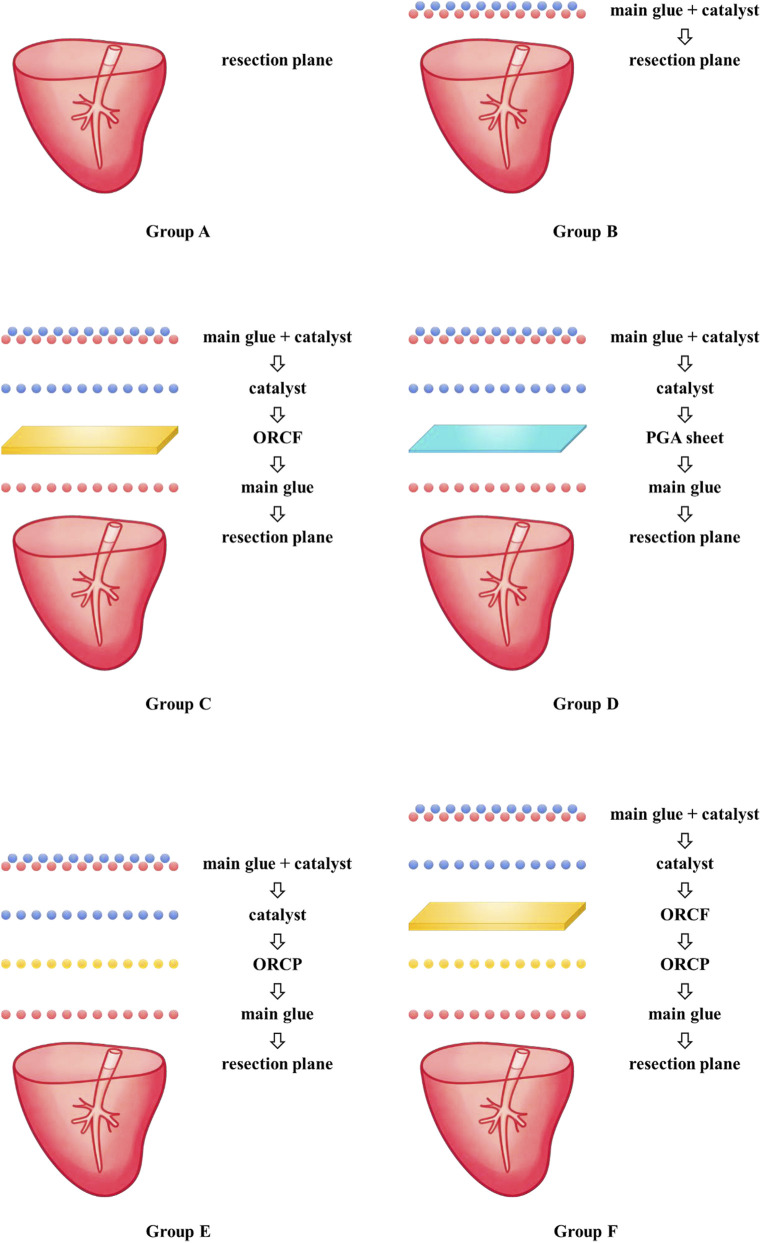
The MALP of the resection planes in each experimental group. Scattered points in the figure denote the measured MALP values of individual samples, and horizontal lines represent the mean value of each group. Group comparisons showed that group A had the lowest mean MALP level overall. The mean MALP of group D was significantly higher than that of group C (p < 0.001). No statistically significant difference in MALP was detected between group D and group E (p = 0.992). In addition, group F had a significantly higher mean MALP than group E (p < 0.001), and presented the highest mean MALP value across all groups.

The MALP values measured at the resection planes were as follows: Group A (control; n = 20): 17.2 ± 1.4 cmH_2_O, Group B (FG; n = 20): 29.1 ± 2.1 cmH_2_O, Group C (ORCF + FG; n = 20): 41.4 ± 3.4 cmH_2_O, Group D (PGA sheet + FG; n = 20): 68.9 ± 4.3 cmH_2_O, Group E (ORCP + FG; n = 20): 68.2 ± 3.2 cmH_2_O, Group F (ORCP + FG + ORCF; n = 20): 101.5 ± 5.2 cmH_2_O. Welch’s ANOVA revealed a statistically significant difference in MALP among the six groups (W = 1904.524, p < 0.001). Post hoc pairwise comparisons using the Games-Howell test indicated that MALP levels in Group F were significantly higher than those in the other five groups (all p < 0.001). No significant difference was observed between Group D and Group E (p = 0.992), but both groups exhibited significantly higher MALP levels compared to Group C (all p < 0.001) (Figure 4).

### 3.2 Histopathological assessments of the porcine lung segmentectomy models

**FIGURE 5:**
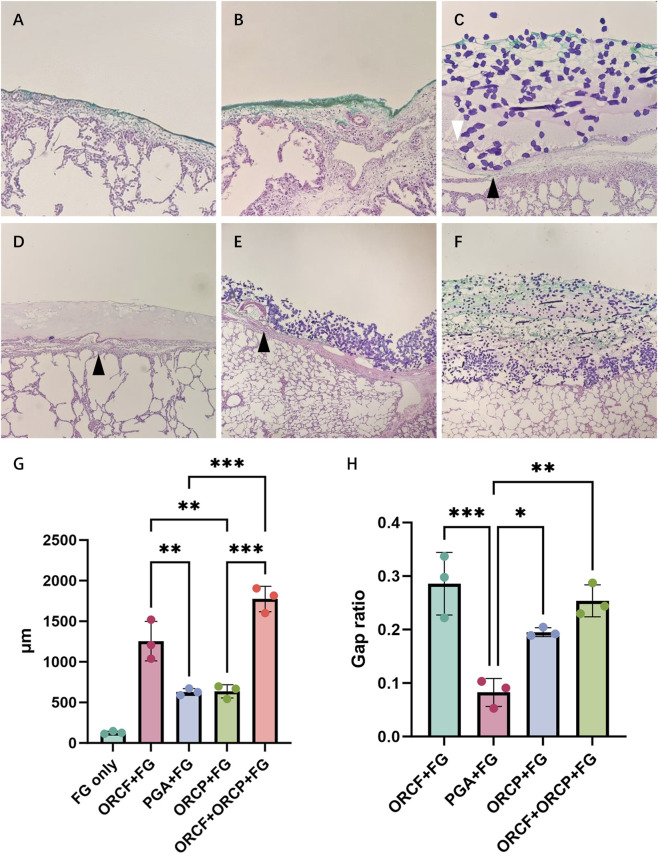
Histological manifestations of the sealing materials on the resection planes. **(A)** Group A: control group, hematoxylin-eosin staining; **(B)** Group B: FG, hematoxylin-eosin staining; **(C)** Group C: ORCF + FG, hematoxylin-eosin staining; **(D)** Group D: PGA sheet + FG, hematoxylin-eosin staining; **(E)** Group E: ORCP + FG, hematoxylin-eosin staining; **(F)** Group F: ORCP + FG + ORCF, hematoxylin-eosin staining. **(A)** The segmental resection plane separated by electrocauterization; **(B)** The glue was unevenly distributed on the surface of the lung tissue, and the overall thickness was less than that of the other experimental groups. **(C)** The sealing layer adhered to the resection surface firmly, and some black gel-like substances accumulated at the pleural defect (black arrowhead). A few gaps could be seen between the sealing layer and the pleura, as well as between the fibers of the ORCF (white arrowhead). **(D)** The sealing layer was tightly adhered to the resection surface and conformed precisely to the uneven regions of the pleura (black arrowhead). **(E)** The sealing layer was tightly adhered to the resection surface and conformed precisely to the uneven regions of the pleura (black arrowhead), with many black gel-like substances distributed within. **(F)** The overall thickness of the sealing layer exceeded that of the other groups, and it closely adhered to the pleura. A large amount of black gel substances was distributed, and the closer to the pleura, the denser the distribution. **(G)** Quantitative analysis of sealing layer thickness in each experimental group. **(H)** Quantitative analysis of the interfacial gap ratio in each experimental group. * Means *p*<0.05; ** means *p*<0.01; *** means *p*<0.001.

To microscopically assess adhesive integrity and tissue response, histological examinations were performed on specimens from each experimental group. Hematoxylin and eosin (H&E) staining of porcine lung tissue revealed:

Figure 5A depicts the segmental resection plane separated by electrocauterization. Figure 5B shows that the glue was unevenly distributed on the lung tissue surface, with its overall thickness being thinner than that of other experimental groups. In Figure 5C, the sealing layer adhered tightly to the resection surface, while some black gel-like substances accumulated at the pleural defect. Meanwhile, minor gaps were visible both between the sealing layer and the pleura and among the fibers of the ORCF. Figures 5D,E both illustrate a sealing layer that tightly adhered to the resection surface and precisely conformed to the pleural unevenness. In addition, Figure 5E shows that abundant black gel-like substances were present within the sealing layer. Figure 5F presents a sealing layer with greater overall thickness than that of other groups; it not only adhered closely to the pleura but also contained a large amount of black gel-like substances, which were more densely distributed in areas closer to the pleura.

We further quantified the thickness of the sealing layer and the results are shown in Figure 5G. The PGA + FG and ORCP + FG groups exhibited relatively thinner sealing layers, whereas the groups containing ORCF showed greater overall thickness. When considered together with the MALP results, these findings suggest that sealing layer thickness alone was not directly associated with mechanical sealing performance across different materials. In addition, the interfacial gap area ratio within the sealing layer was measured, and the results are presented in Figure 5H. The PGA group showed the lowest gap area ratio (0.082 ± 0.02), whereas the ORCF + ORCP + FG group, which exhibited the strongest mechanical sealing performance, still showed a gap area ratio of 0.254 ± 0.02. Taken together, these results suggest that neither sealing layer thickness nor gap area ratio alone can fully explain the differences in mechanical sealing performance among the tested materials.

## 4 Discussion

In recent years, combining PGA sheets with FG to seal lung resection surfaces has become a common clinical method for preventing postoperative pulmonary air leakage (Suzuki et al., 2022). However, its wide application is limited by several factors.

These include relatively high costs and limited accessibility in certain clinical settings, which can increase the economic burden on patients. Furthermore, previous studies have reported that PGA-based sealants may induce robust inflammatory responses and dense pleural adhesions due to the degradation product glycolide. These fibrotic sequelae can significantly increase the complexity and risk of future ipsilateral reoperations (Nakamura et al., 2010; Saito et al., 2017). In contrast, ORC-based materials are generally more accessible and cost-effective; their long-term tissue response in this sealing application requires further validation.

This has led to the difficulty in promoting the method of combining multi-layer PGA sheets and FG in clinical practice (Nomori et al., 2014).

To address these limitations, our center has actively explored more effective strategies. We focus on using FG combined with alternative materials after pulmonary resection to prevent air leakage. In previous studies, we investigated Surgicel® Absorbable Hemostat. It is a commonly used hemostatic agent and its main component is ORC, which has been investigated as an adhesion-reduction material in previous studies (Ten Broek et al., 2014; Kanai et al., 2020). It has also been reported to have favorable tissue compatibility in other settings (Ebana et al., 2018). We established an *ex vivo* porcine lung segmentectomy model in order to evaluate the anti-air-leakage effect of ORCF combined with FG. Results showed that the MALP in the ORCF group was 41.8 ± 4.5 cmH_2_O. In the PGA group, the MALP was 69.5 ± 5.2 cmH_2_O. Both values exceed the clinically expected intrathoracic pressure range of 30–40 cmH_2_O in the early postoperative period (Araki et al., 2007), which indicates acceptable sealing ability. Nevertheless, there is still a difference in sealing effectiveness between ORCF and PGA sheets. Furthermore, all experiments were performed on healthy, excised porcine lungs. For patients with impaired lung tissue (e.g., emphysema patients) or under conditions of sudden increases in airway pressure (caused by coughing, mucus plugging, or bronchospasm), additional studies are needed to determine whether ORCF can achieve similar effects to PGA sheets.

SURGICEL Powder is a granular hemostatic agent produced by compressing fine ORC fibers into a porous, particulate structure. This unique physical configuration enables close conformity to irregular wound surfaces and enhances adherence to bleeding sites (Al-Attar et al., 2023), while retaining the inherent biocompatibility, biodegradability, and low toxicity of ORC. Clinical evaluations have shown no evidence of granuloma formation at implantation sites within 28 days postoperatively (MacDonald et al., 2017). Given its structural advantages, we hypothesized that the aggregated granular form of ORC could provide superior sealing on the uneven surfaces resulting from pulmonary segment resection, when used in conjunction with FG. To test this hypothesis, we employed the same experimental methodology as in our previous study and compared various sealing approaches. The results demonstrated that the MALP in the ORCP + FG group was 68.2 ± 3.2 cmH_2_O, while the PGA sheet + FG group achieved 68.9 ± 4.3 cmH_2_O. No statistically significant difference was observed between these two groups, both of which were significantly higher than the ORCF + FG group (41.4 ± 3.4 cmH_2_O). These findings suggest that ORCP, when combined with FG, offer superior air-leak resistance compared to ORCF and achieve a sealing efficacy comparable to that of PGA sheets. Notably, the composite approach using ORCP + FG + ORCF yielded a MALP of 101.5 ± 5.2 cmH_2_O, significantly exceeding all other groups.

While a baseline burst pressure of >40 cmH_2_O is typically required for standard sealing materials under resting conditions, the exceptionally high MALP (>100 cmH_2_O) achieved by the ORCP-based strategy may be clinically relevant in the context of transient postoperative pressure spikes. During the early postoperative recovery phase, patients frequently experience transient yet extreme airway pressure spikes. Notably, in addition to forceful coughing, routine clinical care procedures such as endotracheal suctioning can also provoke abrupt and severe surges in intrathoracic pressure, which can readily approach or exceed 60–100 cmH_2_O (McCool, 2006). A sealant that merely meets the basic resting pressure requirements remains vulnerable to blowout during these events. The substantial pressure-resistance demonstrated in our study provides a critical safety margin. This robust mechanical integrity may help maintain seal integrity under acute physiological stress; however, its potential effects on delayed air leaks, chest tube duration, and postoperative recovery require future *in vivo* and clinical validation.

Histological examination (Figure 5) showed the following results. In Group C, the ORCF and FG layer adhered to the resection surface. Scattered deposits of black gel-like substances were present in the sealing matrix. These structures correspond to acid-heme complexes. The complexes are formed by the reaction between carboxyl groups in cellulose and Fe^2+^ ions from blood components (Wu et al., 2012). The black gels are embedded in the composite sealing layer consisting of ORCF and FG and tend to accumulate at pleural defect sites. Based on our previous findings, it can be inferred that these complexes contribute to the filling of pleural defects. However, minor voids persist between the sealing layer and the pleura, as well as among the fibers of the ORCF, which may not have been fully occupied. This incomplete integration may be related to the capillary absorption of fibrin gel by the ORCF (Wu et al., 2012).

In contrast, the sealing layers in Group D, E, and F adhered closely to the resection surfaces. This enhanced apposition is likely due to two factors. One is the fluidity of the FG. The other is the fluidization phenomenon that occurs when ORCP are incorporated into the adhesive matrix. Under such conditions, the particles act like a fluid. This allows the sealant to fully conform to irregular pleural surfaces. To strengthen the objectivity of tissue-integration assessment, quantitative histological analysis was performed. The PGA + FG group exhibited the lowest interfacial gap ratio, suggesting improved sealing interface conformity. In comparison, the sealing layer thickness in the ORCP + ORCF + FG groups showed minimal change relative to ORCF + FG, whereas the interfacial gap ratio was significantly reduced after ORCP application. These results quantitatively support the enhanced sealing performance observed with combined-material approaches. The sealing layers in Group E and F showed dense distribution of black gel-like substances. These substances are acid-heme complexes generated by the contact between ORC material in the aggregate particles and blood. This is similar to the complexes observed in Group C. Notably, in Group F, the combined layer (ORCP, FG, and ORCF) had the greatest overall thickness, and there was no distinct interface between individual components. Meanwhile, the layer adhered tightly to the pleural surface. Histological analysis suggests that ORCP may integrate with FG. This integration may contribute to the sealing efficacy of the fibrin matrix and improve the bonding strength of the overlying ORCF.

In this study, we used the established application technique for sealing materials described by Gika et al. (2007), Itano (2008). Our prior method involved direct placement of an ORCF after spraying fibrinogen solution. By contrast, the approach in Group F enables ORCP to penetrate the fibrinogen layer and react directly with blood. This reaction forms black gel-like clots that seal pleural defects. After being soaked in blood, ORCP will also expand due to the loosening of the connections between their fibers. During this process, some aggregate particles will separate into fibers (Wang et al., 2017).

Application of multiple batches of ORCP results in their overlap. Through fiber separation, they form an interconnected, network-like structure within the FG. This structure has superior flexibility compared to sheet materials while maintaining adequate tensile strength. Then, ORCP saturated with fibrinogen solution interact with ORCF saturated with thrombin solution. This interaction induces extensive fibrin clot formation throughout the network. Furthermore, ORCP are widely dispersed across the wound surface, which provides numerous anchoring points, facilitating firm fixation of the overlying sheet. Additionally, unlike rigid sheet materials (with fixed dimensions and shapes), sprayed ORCP show excellent adaptability to larger and more irregular resection surfaces. They effectively fill tissue defects and create a relatively smooth interface for subsequent sheet application. Histological features observed in Group F are consistent with this proposed mechanism, which requires further validation. ORC particles also have several advantages: low cost, complete biodegradability, and minimal toxicity of degradation products (Al-Attar et al., 2023; Gika et al., 2007). These properties suggest their potential as an alternative to PGA sheets, pending further validation.

A limitation of this study is the use of an acute, excised healthy porcine lung model. Emphysematous lungs were deliberately excluded to enable a standardized baseline comparison of the intrinsic mechanical performance of different materials without the confounding effects of extreme parenchymal fragility. Furthermore, because this *ex vivo* model lacks active physiological dynamics such as blood perfusion, ventilation, and immune responses, long-term inflammatory response, fibrosis, and adhesion formation could not be assessed.

This study was motivated by the persistent clinical challenge of postoperative air leaks following pulmonary segmentectomy and evaluated hemostatic materials already widely used in clinical practice. Accordingly, the investigation was designed as a controlled *ex vivo* mechanical comparison to isolate intrinsic sealing performance. Previous studies have shown that improvements in mechanical sealing strength alone can translate into meaningful clinical benefits, including reduced postoperative air leakage and shorter chest tube duration.

Although these clinically registered materials have established safety profiles, identifying strategies that combine strong mechanical sealing with minimal adverse biological responses remains our primary goal. Therefore, future *in vivo* survival studies, including elastase-induced emphysematous models, are warranted to evaluate long-term inflammatory response, tissue integration, and adhesion formation, and to further validate the clinical applicability of this ORCP-based sealing strategy. Accordingly, conclusions regarding long-term tissue response and clinical outcomes should be interpreted cautiously.

## 5 Conclusion

Compared with the combination of PGA sheets and FG for preventing air leakage after pulmonary segmentectomy, ORCP may offer practical advantages such as lower cost and accessibility, whereas long-term toxicity and adhesion formation require further *in vivo* validation. When used in combination with ORCF and FG, they significantly enhance mechanical air-leak sealing in this *ex vivo* model. This material, along with the pleural coverage technique, demonstrates strong mechanical sealing capability and supports further *in vivo* and clinical evaluation.

## Data Availability

The raw data supporting the conclusions of this article will be made available by the authors, without undue reservation.
